# Are Coastal Protected Areas Always Effective in Achieving Population Recovery for Nesting Sea Turtles?

**DOI:** 10.1371/journal.pone.0063525

**Published:** 2013-05-03

**Authors:** Ronel Nel, André E. Punt, George R. Hughes

**Affiliations:** 1 Department of Zoology, Nelson Mandela Metropolitan University, Port Elizabeth, South Africa; 2 School of Aquatic and Fishery Sciences, University of Washington, Seattle, Washington, United States of America; 3 Retired Conservator, Howick, South Africa; Monash University, Australia

## Abstract

Sea turtles are highly migratory and usually dispersed, but aggregate off beaches during the nesting season, rendering them vulnerable to coastal threats. Consequently, coastal Marine Protection Areas (MPAs) have been used to facilitate the recovery of turtle populations, but the effectiveness of these programs is uncertain as most have been operating for less than a single turtle generation (or<20 yr). South Africa, however, hosts one of the longest running conservation programs, protecting nesting loggerhead (*Caretta caretta*) and leatherback (*Dermochelys coriacea*) turtles since 1963 in a series of coastal MPAs. This provides a unique opportunity to evaluate the long-term effect of spatial protection on the abundance of two highly migratory turtle species with different life history characteristics. Population responses were assessed by modeling the number of nests over time in an index area (13 km) and an expanded monitoring area (53 km) with varying survey effort. Loggerhead abundance increased dramatically from∼250 to>1700 nests pa (index area) especially over the last decade, while leatherback abundance increased initially∼10 to 70 nests pa (index area), but then stabilized. Although leatherbacks have higher reproductive output per female and comparable remigration periods and hatching success to loggerheads, the leatherback population failed to expand. Our results suggest that coastal MPAs can work but do not guarantee the recovery of sea turtle populations as pressures change over time. Causes considered for the lack of population growth include factors in the MPA (expansion into unmonitored areas or incubation environment) of outside of the MPA (including carrying capacity and fishing mortality). Conservation areas for migratory species thus require careful design to account for species-specific needs, and need to be monitored to keep track of changing pressures.

## Introduction

Marine Protected Areas (MPAs) may be one of the most effective tools for biodiversity conservation [1], [2], but are somewhat controversial for fisheries management as they tend to displace rather than reduce fishing effort [3]–[5]. Highly mobile species may also benefit only marginally if only a part of the life cycle is protected in MPAs. Definitive direct evidence on the effectiveness of protected or closed areas leading to the recovery of highly migratory marine species, such as sea birds, whales, sharks, pelagic fish or sea turtles, is thus scarce, even though spatial measures are often suggested as a conservation tool [6]–[9]. Much of the current literature on migratory species focuses on their spatial distribution [10]–[15], especially on areas of high concentration and preferred habitats [15]–[17], threats such as fisheries overlapping with their spatial distribution [18], effects of habitat destruction (pollution, ghost fishing and feral pests) [19], genetic stock identification [10], [20], or potential options and considerations for conservation. Population recovery/decline is usually a result of changes in the abundance of trophic competitors [15] or a reduction/increase in fisheries impacts [21], [22]. Sea turtles provide good case studies to evaluate the success of spatial conservation efforts in migratory species applied in multi-species settings and where they have been maintained over time [23].

The suite of threats faced by sea turtles range from targeted harvesting of females or eggs on the nesting grounds through incidental deaths during fisheries activities [24], [25] to habitat degradation or destruction [26]. The relative importance of these threats depends on species, location, life history phase or the size of the rookery [27], [28]. Rookeries may be particularly vulnerable, because several life history phases are present at high densities (eggs, newly hatched or neritic sub-adults, adults during the breeding migration and inter-nesting periods) [29], and thus tend to be easy prey for predators and harvesters. Consequently, conservation may be most effective on the coast where sea turtles occur in large aggregations during sensitive stages of their life history and where combinations of threats can be eliminated [26].

**Figure 1:**
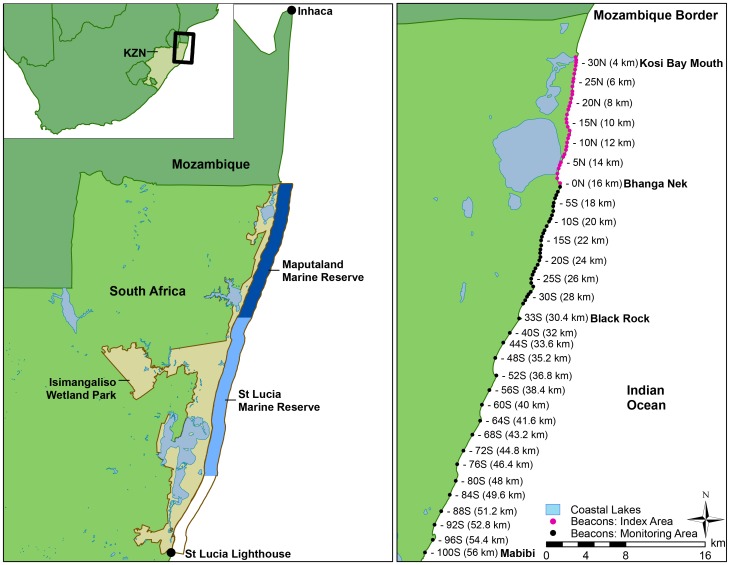
Turtle nesting areas in South Africa, and study area for the long-term monitoring program, indicating the marine reserves, index and monitoring areas for the 56 km south of the Mozambique border.

Conservation actions typically involve implementing nest protection programs, or formally proclaiming coastal or marine protected areas, which may include both inter-nesting and nesting habitat [29]. These measures restrict human access to sea turtles, while also protecting nesting and inter-nesting habitat. Examples of recovering populations as a result of some form of coastal/marine protection include the green turtles (*Chelonia mydas*) of Aldabra [30], Grande Glorieuse and Europa Islands [31], Ascension Island [32] and Hawaii [33], hawksbill turtles (*Eretmochelys imbricata*) from the Cousin and Aldabra islands, Seychelles [34], and leatherbacks from French Guiana/Suriname and Gabon [35], the Caribbean [36] and Florida [37]. One of the most extensive coastal and marine conservation programs has been maintained in South Africa, protecting both nesting and non-nesting sea turtle species, their nesting and some of the inter-nesting habitat in a series of coastal and marine protected areas (Figure 1). Interim reviews of the effectiveness of these conservation measures concluded that the program has been a conservation success [38], [39].

Despite these reviews, there has never been a quantitative review of the two species (controlling for monitoring effort) and their long-term response to conservation. Further, more advanced analytical techniques are now available to model the species responses to management intervention. Thus, nearly five decades after implementation, it is appropriate to review the performance of the physical and spatial protection program on the recovery of loggerheads and leatherbacks nesting in northern KwaZulu-Natal (KZN). The expectation was that both species of sea turtles would benefit from protection, given that they were facing similar threats and that these were addressed through the program. We estimate the recovery potential of the two species by comparing their basic nesting biology, reproductive output and initial nesting numbers. We evaluate the long-term trends from the quantitative monitoring program that spans 4.5 decades of protection and data collection to ascertain (1) if both species benefited from coastal protection, (2) if the benefit was comparable for the two species, taking into account the differing initial numbers and breeding biology, and (3) if the original targets of population growth have been reached. This comparison in population trends between the species should be useful to assess longer term effects of conservation interventions and the response of species with different biology. We also discuss deviations from the predicted population responses.

## Methods

### Ethics statement

All nesting data were collected by the provincial conservation authority (Natal Parks Board later named Ezemvelo KZN Wildlife) in accordance with their legislated conservation mandate. This is one of several long-term population monitoring programs.

### Study Area and History

The South African (SA) turtle rookery is situated in the northeast corner of the country (Figure 1) sharing nesting populations of loggerhead and leatherback turtles with Mozambique, and hawksbill and green turtles with the rest of the Western Indian Ocean. There are no historical records indicating the sizes of these populations before the inception of conservation measures. However, SA has some of the oldest examples of human-turtle interactions. Turtle bone fragments have been found in a human-inhabited cave dating back to the middle stone age [40]. The population effects of use are, however, poorly documented, but it is assumed that the populations were depleted (to some extent) when formal protection of sea turtles started with legislation introduced in 1916. The Natal Coastal Fisheries Ordinance banned the harvesting of sea turtles and eggs [41], [42], but this regulation was poorly enforced. The indigenous ama Thonga people seemed to have developed an interest in utilizing sea turtles, with a history of harvesting eggs as a source of protein [43]. The Natal Parks Board initiated an active field-based protection and monitoring program in 1963, given the growing interest in sea turtle meat [42] and the potential cash value developing around turtle products [43]. After the first seasons of field monitoring, McAllister et al [42] recommended that the nesting beaches should be turned into a turtle sanctuary with restricted human access. The first stretch of beach (the St Lucia Marine Reserve; Figure 1) was proclaimed a marine reserve in 1979. At the time it was recognized that the localized protection did not curtail harvesting of sea turtles outside of the reserve, and certainly not along the east-African coast. The sustainable use aspect, and dependence of poor communities on sea turtles, was recognized, and the spillover effect from the reserve became an explicit objective [43]. The rest of the 150 km stretch of coast to the Mozambique border was formally proclaimed as the Maputaland Marine Reserve (Figure 1) in 1986 to enhance the rate of population recovery, as well as the contribution to ex situ subsistence harvesting [39]. Both these marine reserves were contiguous with a series of terrestrial reserves. The beaches and the offshore coral reefs were also established RAMSAR sites (Convention on Wetlands of International Importance; RAMSAR site No 344 Turtle Beaches/Coral Reefs of Tongaland), which highlights their regional importance in terms of biodiversity. At the time, the objective was local protection of non-nesting species, nesting females and their nests/eggs, and the habitat. If the populations recovered fast enough or to a sufficient size, local harvesting would have been reconsidered [43]. An arbitrary population (size) target was set at∼200 leatherback and 500 loggerhead nesting females per annum. However, given the global declines in sea turtle populations, especially leatherbacks [44], the original objectives changed and conservation became the priority. The area achieved the highest conservation accolade in 1999, when the beaches (with rocky shores, mangroves, lakes and estuaries) and coastal waters to three nautical mile (5 km) offshore were proclaimed a United Nations Educational, Scientific and Cultural Organization's (UNESCO) World Heritage Site (now known as the iSimangaliso Wetland Park, Figure 1). These regulations provided near complete habitat protection to 200 m depth, including the coral reefs, restricting activities to non-consumptive scuba diving on some reefs and limited pelagic recreational fishing. Commercial or industrial fishing was completely excluded, as was any kind of coastal development or pollution. Today, conservation protection continues, poaching is incidental, and tourism and conservation partnerships have developed with the local community. In 2009, the protection expanded further when Africa's first across-border marine conservation area with Mozambique (the Ponto du Ouro – Kosi Bay Transfrontier Marine Conservation Area) was proclaimed.

### Field sampling

Since the Maputaland program is one of the first turtle monitoring programs in the world, some methodological experimentation took place during the early years involving tagging methods, patrol distance and frequency. However, survey effort and protocols were well-documented in annual season reports, and therefore could be considered in the analyses (Table 1). All methods have been standardized since 1973.

**Table 1:** Monitoring protocol over time for the index and monitoring areas.

Monitoring Season (Years)	Distance patrolled (km)*	Method and Dates of Patrols	Tags applied and carapace metrics obtained	Reference documenting effort and protocols
1965/66–1972/73	Index area only (12.8 km)	Vehicle patrols from October; Intensive foot patrols during peak nesting (December & January)	Plastic tags (ROTO/ORI)**CCL for both species	[42],[45]–[47]
1973/74–2009/10	Monitoring area (including the index area)	18 October to 20 March of each year; using vehicle and foot patrols	Monel/Titanium **SCL for Cc and CCL for Dc	[48]

* Index area: 3.2–16 km south of the border; Monitoring area: 3.2–56 km south of the border.

** Curved carapace length  =  CCL & Straight carapace length  =  SCL.

Data collection entailed nightly patrols every austral summer between mid-October and mid-March (defined as a “nesting season”). The species and carapace size of each sea turtle encountered were recorded. Each female turtle was also tagged with a flipper tag. The tag was placed at the proximal end of the front flippers for loggerheads, while the back flippers were used for leatherbacks. When sea turtles emerged without being observed, tracks were scored as either “nested” or “not nested”, and counted with the relative position along the beach noted. Tracks were scored as “nested” if a clear body pit was excavated and sand was disturbed over body pit and track as predator disguise. All other track and dig attempt configurations were scored as “not nested”. Morning patrols at sunrise recorded all unreported tracks/nests of turtles that emerged after nightly monitoring ceased.

The data for the first eight years (1965–1972) were collected with consistent, but restricted, effort from the research station (16 km south of the Mozambique border) to the Kosi Estuary mouth (3.2 km south of the border) (Figure 1). Patrolling of this area has taken place every season since 1965, and hence is the “index area” (12.8 km), with data for 1965/66–2009/10. The survey area was expanded after 1972/73, permanent markers (beacons 30N–100S; Figure 1) were erected and the patrolling extended to 56 km south of the border. The “monitoring area” is thus from 3.2 km to 56 km ( = 52.8 km) south of the border and has been monitored consistently from 1973/74 (Table 1; Figure 1).

### Population Trends and Spatial Distribution

The position of each turtle nest was recorded in relation to a fixed point (beacon) along the beach, with 400 m accuracy in the high density area, or 1 600 m (1 mile) accuracy in the lower density area (Figure 1). Accuracy of 400–1600 m is sufficient considering that the monitoring area is∼53 km, and the total length of beach used by sea turtles for nesting in South Africa exceeds 150 km with an additional, continuous strip used for nesting across the border into Mozambique [49], [50].

The two data sets (index and monitoring) were used to determine if there was a significant change in the number of nests over time for each species. These data sets respectively cover the monitoring area of∼53 km (1973/4–2009/10) and∼13 km (1965/66–2009/10) (Figure 1). The index area includes a dense concentration of loggerheads, but under-samples leatherbacks, which seem to have weak spatial preference and are known to nest outside of the monitored area (RN, pers. obs). The two data sets (index and monitoring areas) were analyzed using Generalized Additive Models, GAMs [51]. GAMs (using R ver. 2.12) were preferred for this study over Generalized Linear Models due to their ability to model a response variable as a non-linear function of covariates. A number of analyses were conducted to examine the robustness of the inferences regarding trends over time. The simplest model assumed that the number of nests was related to nesting season (season *y* defined as October 18 of year *y* to March 20 of year *y*+1) by means of a regression spline, with the number of knots selected using general cross validation, i.e.: 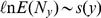
(1) where 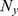 is the number of sea turtles observed during the nesting season *y*. The observed numbers of nests were assumed to be negative binomially distributed because initial attempts to fit model (1) assuming a Poisson distribution indicated substantial over-dispersion. No account was taken of effort in Equation 1 because effort was controlled for in the design of the index and monitoring data sets (as per Table 1).

The alternative models were based on a data set, which consisted of the numbers of observed nests by season, week within season, and distance in steps of 400 m (index data set) and 2 km (monitoring data set). Models were considered in which season, week and distance were modeled using regression splines, i.e. 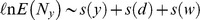 The interaction between each combination of covariates was also modeled using a tensor spline (with either 40 or 100 knots to define the 2-d splines). The data set on observed nests was augmented by zeros when no nests were observed for a given combination of year, distance and week so that the data set was fully balanced. The alternative models were fitted assuming that the response variable was Poisson distributed as there was no evidence for over-dispersion when the data were disaggregated spatially and temporally. The resulting model fits were evaluated using q-q plots of the deviance residuals, as well as plots of residuals against the fitted values and the covariates.

The model outputs were used to indicate average spatial and within-year patterns in nesting as well as to compare preferences between species, and to examine whether changes over time have occurred. The premise of the conservation plan is that the two species will show similar responses over time, assuming that the same pressures are eliminated. The predicted numbers of nests per species in the monitoring area were used to determine the relative abundance (as a ratio) between the two species over time.

### Reproductive Output

Reproductive output (as a proxy for recovery potential) for females per species was estimated from the inter-nesting interval, the number of nesting events per species per season, and the remigration period between seasons using the data for the 1965/66 to 2009/10 seasons. The potential number of hatchlings produced per season was calculated using the mean number of eggs per nest and emergence success (hatchlings that emerge above the surface relative to the number of eggs in the nest) from Hughes [50] and the number of nests per season produced by each species. The inter-nesting interval (i.e. the period, in days, between successive nesting events within a season) for each species was obtained by calculating the number of days between consecutive sightings of tagged individuals. These data were modeled under the assumption that the inter-nesting interval is normally distributed among individuals and independent among nesting events, and that some nesting events are missed. The distribution of the inter-nesting intervals was therefore estimated by fitting a model in which the observed time between nesting events was a mixture of normal distributions, i.e. the likelihood of an observed inter-nesting time *T* is:
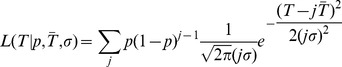
(2) where *p* is the probability of observing a nesting event given it has occurred, 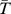 as the mean inter-nesting interval, and 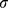 is the among-individual variation in within-year nesting interval. The first element of the likelihood function (*j* = 1) is the probability of observing the next nesting event, while the 2^nd^ element (*j* = 2) is the probability of observing a nesting event given that one nesting event was missed. The 3^rd^ and subsequent elements are defined analogously. The data used to fit Equation 1 were restricted to inter-nesting intervals of 4–200 days. Data on inter-nesting intervals of 1–3 days were ignored because these inter-nesting intervals were probably a reflection of disturbance during an earlier nesting event and half clutches laid on consecutive days.

## Results

### Population Trends

A total of 108 878 loggerhead and 14 607 leatherback emergences (or tracks) were recorded over the 45 years of monitoring. Of these emergences, 60 946 loggerheads nested and 47 932 were reported as tracks only (i.e., tracks that did not result in a nest). A total of 38 052 loggerhead turtles were handled, allowing for tags to be read and individual size to be measured (straight carapace length in mm, SCL). The numbers for leatherbacks were: 13 320 nested, 1 287 tracks only, with 5 304 individuals tagged and measured (as curved carapace length in mm, CCL). Loggerhead turtles therefore nested only half (55%) of the time they emerged, whereas leatherbacks nested nine out of ten times (or 91%). 12 774 individual turtles were identified from over 38,000 encounters (with a mean±SD SCL of 860.4±47.5 mm, n = 13 109), with a mean number of 371.1±104.9 (mean±SD) individuals per season. More than 78% of these individuals were seen for one season only and 15% for a second season (Table 2). The maximum reproductive lifespan was around 18 years (Table 2). In contrast, 86% of leatherbacks nested for one season only and 8.5% for a second season, and the typical maximum reproductive lifespan was about 16 years (and 19 as the exception; Table 2). 2 578 unique individuals were identified in the 5 304 encounters (1604.6±101.6 mm mean±SC CCL, n = 2 235), with a mean number of individuals per season at 69.4±38.1. On average, five times fewer leatherbacks were handled than loggerheads.

**Table 2:** Tag resighting information (no of seasons, reproductive lifespan and remigration period) for loggerhead and leatherback turtles in Maputaland.

Species	No of seasons sighted	No (%) of individuals	Reproductive lifespan (yrs)	Mean remigration period (yr)
Loggerheads	1	10053 (78.7%)	NA	
	2	1935 (15.1%)	2–37* yrs; Mode = 3	2.2
	3	533 (4.2%)	3–18 yrs; Mode = 5	2.3
	4	150 (1.2%)	4–27* yrs; Mode = 7	2.2
	5	63 (0.5%)	6–18 yrs; Mode = 9	2.1
	6	26 (0.2%)	8–18 yrs; Mode = 12	1.9
	7	6 (0.05%)	12–15 yrs; Mode = 14	2.0
	8	5 (0.04%)	14; 17; 15; 11; 8	1.6
	9	2 (0.02%)	11; 12	1.3
	10	1 (0.01%)	14	1.4
n =		12 774		
Leatherbacks	1	2231 (86.5%)	NA	
	2	220 (8.5%)	2–16 yrs; Mode = 3	2.2
	3	76 (2.9%)	3–15 yrs; Mode = 6	2.4
	4	35 (1.4%)	6–16 yrs; Mode = 9	2.4
	5	12 (0.5%)	10–19 yrs; Mode = 10	2.7
	6	3 (0.1%)	12; 14; 11	2.0
	7	1 (0.04%)	12	1.7
n =		2 578		

* Potential tag confusion due to similar tag codes e.g. E274 vs EE274; next longest breeding duration value = 18 years (max).

**Figure 2:**
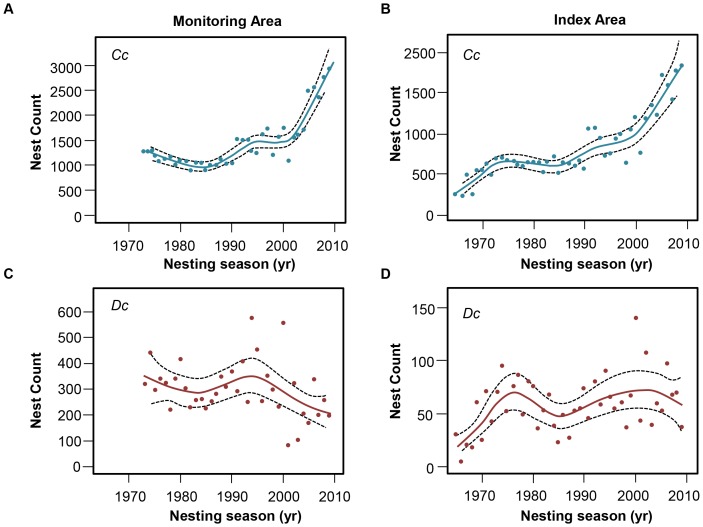
Counts and baseline model-estimated time-trajectories of counts for loggerheads (Cc; A&B) and leatherbacks (Dc; C&D) for the monitoring area (left panels) and index area (right panels).

Of the models including distance and week covariates, the model with a year*distance interaction and week as a covariate was selected using Akaike's Information Criterion, AIC. Results are therefore shown for the baseline model [s(year)], a model with smoothers for week, year and distance from the border, and a model with smoothers for distance*year and week. The predicted trends in number of nests from three models were consistent (Figure S1) and the fit of the model (e.g. baseline model, s(year)) to the data was good (Figure 2). There was a marked (and significant) increase in the number of loggerhead nests over time, especially in the last decade, irrespective of the choice of data set (Figure 2A&B). In contrast, the trend in the number of leatherback nests differed depending on whether the analysis was based on the monitoring area or the index area, with a decline since 1994 for the monitoring area (Figure 2C) and an oscillating, but stable, pattern for the index area (Figure 2D).

**Figure 3:**
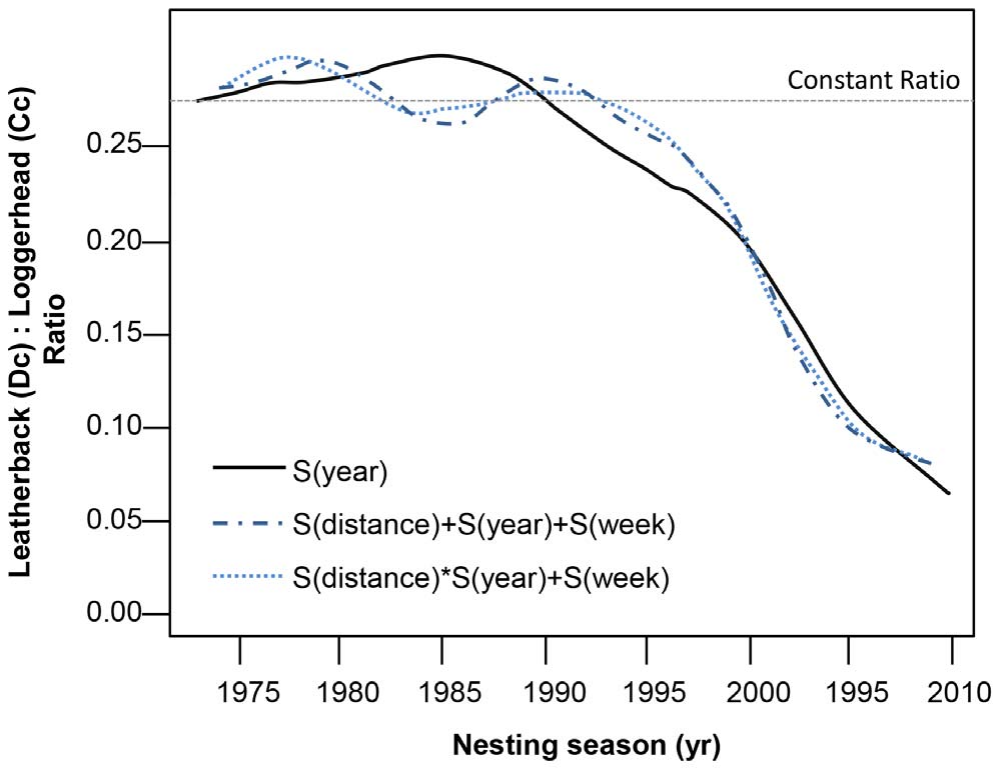
Ratio of the number of leatherback to loggerhead nests over time for the monitoring area from three of the GAM models fitted to the nesting count data.

The ratio between the two species changed considerably during the monitoring period, starting from a ratio of 0.27∶1 Dc:Cc (Figure 3, straight dotted line). However, leatherbacks increased faster during the first 15 years, but then declined relative to loggerheads, and were below the initial ratio 0.27∶1 by 1990. Since then, the number of loggerheads has increased dramatically compared to the number of leatherbacks (Figure 2). The rates of change were thus not constant over time and suggest that the populations did not respond as expected. This raises the question as to the causes of relative success, which may be inherent differences in biology, changes in incubation environment or pressures/opportunities further afield (i.e. different foraging habitat quality or offshore pressures).

### Spatial Distribution

**Figure 4:**
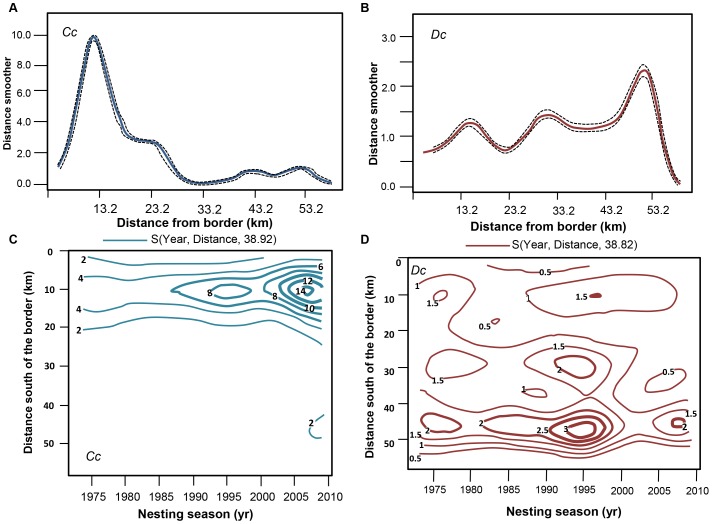
Model-estimated indices of relative abundance for the monitoring area by distance from border from the model s(year)+s(distance)+s(week) (upper panels), and the interaction between distance and year from the model s(year * distance) + s(week) (lower panels). Results are shown for loggerheads (*Cc*; A&C) and for leatherbacks (*Dc*; B&D).

The model-predicted distribution of the density of nests along the monitoring area differed substantially between the two species (Figure 4). Loggerhead nesting was concentrated in the northern half of the monitoring area, peaking 10–16 km south of the (Kosi) estuary mouth (Figure 4A&C). This preferred area remained constant, even as the population was increasing. The density of the contours became tighter, with a minor expansion into marginal areas at 45 km south, as the loggerhead population increased in size (Figure 4C). Leatherbacks, on the other hand, showed less spatial preference, with nests distributed along the entire monitoring area. Three temporally stable preferred areas could be identified, with nesting concentrated around 50 km south of Kosi estuary mouth (Figure 4B&D).

### Reproductive Output

**Figure 5:**
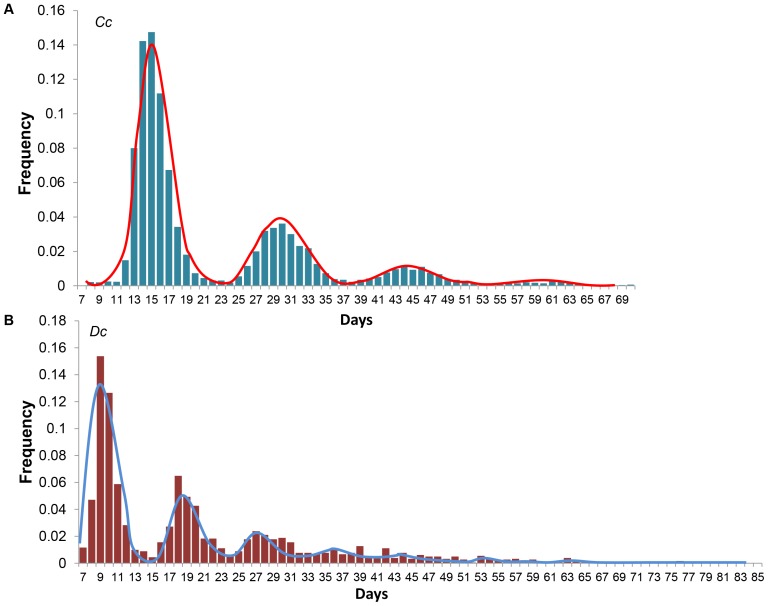
Observed distributions of the within-season inter-nesting interval in days (bars) and the fit of the model to those data (solid line) with A) loggerheads (***Cc*** **) and B) leatherbacks (** ***Dc*** **).**

There was a marked difference between species in the number of nests per season (Figure 5). The majority (92%, n = 138) of loggerhead turtles were observed to nest 3–5 times per season (3.7±0.8 times; mean±SD), and 79% (n = 43) of leatherbacks were observed to nest 6–8 times (6.7±1.5; mean±SD) per season. The model (Equation 2) fitted the data well (Figure 5), with multiple modes clearly evident for both species. The mean inter-nesting interval was 9.5 days (SE = 0.034) for leatherbacks and 15.0 days (SE = 0.02) for loggerheads. The among-individual variation in inter-nesting interval was larger for loggerheads (σ = 2.18; SE = 0.019) than for leatherbacks (σ = 1.40; SE = 0.027). The model also provided an estimate of the probability of observing nesting events per species. Even though loggerheads nested fewer times than leatherbacks, the probability of observing a loggerhead nesting event (0.67, SE = 0.01) was higher than that of observing a leatherback nesting event (0.46, SE = 0.01).

**Figure 6:**
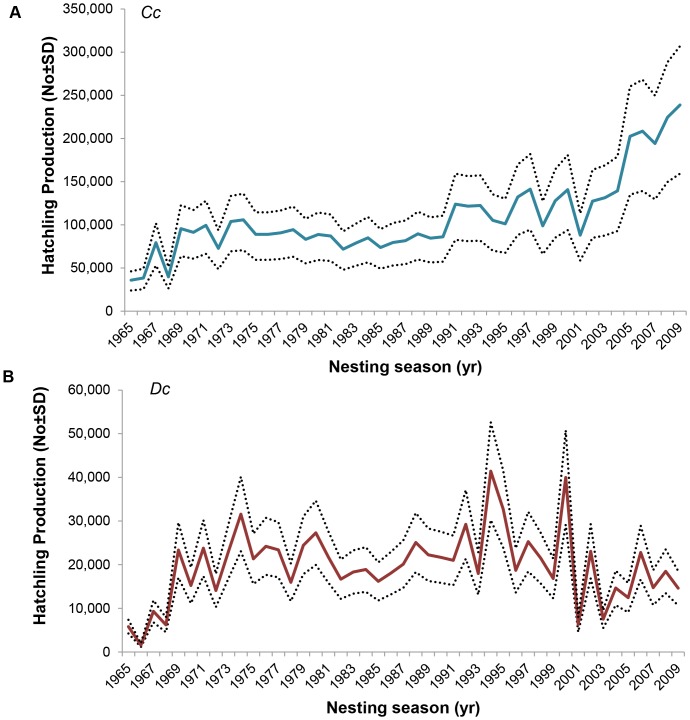
Estimated hatchling production for A) loggerhead (*Cc*) and B) leatherback (***Dc***) turtles in Maputaland over time (with the mean±SD) presented.

Due to the higher nesting frequency, leatherbacks had a higher reproductive output per individual female over the season. Both species laid similar number of eggs per nest (as reported by [50]): the mean number of shelled yolked eggs per nest was 105 (n = 72, range: 39–154) and 104 (n = 39, range: 55–142) for leatherbacks and loggerheads, respectively. Emergence success for loggerheads was 77.8% (n = 72, SD = 25.9%) and 68.9% (n = 39, SD = 18.6%) for leatherbacks. Each loggerhead female produced an average of 389 eggs per season, with 302 emerging hatchlings, while leatherbacks females laid 699 eggs per season, with 480 hatchlings emerging. However, the absolute recovery potential as measured by the total number of hatchlings produced was substantially higher for loggerheads than for leatherbacks. Loggerheads produced 63 412–143 842 hatchlings per season (302 hatchlings per female times 371.1(±104.9) females per season) while leatherbacks produced 36 583–51 610 hatchlings (480 hatchlings times 69.4±38.1 females). The hatchling production for the duration of the program across the monitoring area ranged 23 973–318 271 loggerhead hatchlings and 1 171–53 139 leatherback hatchlings depending on the season (Figure 6). The absolute recovery potential of loggerheads has been higher because the absolute number of loggerheads has always been larger.

**Figure 7:**
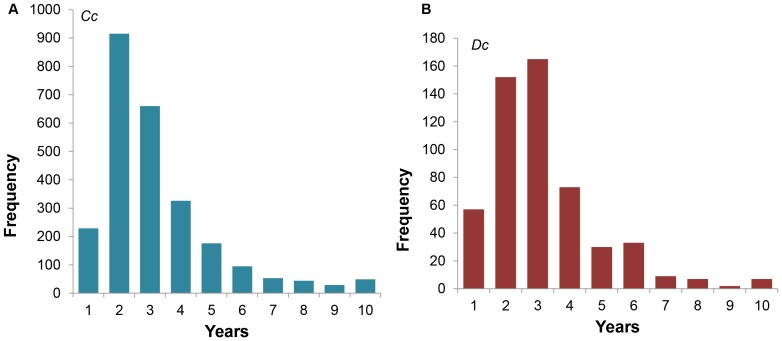
Remigration intervals in years for A) loggerhead (*Cc*) and B) leatherback (*Dc* **) turtles.**

The period between successive nesting seasons, i.e. the remigration period, was very similar between the two species (Mann-Whitney U = 46.5, P = 0.82, n = 20 Statistica ver.9; Figure 7). Loggerhead females returned after 1 109±699 days (or 3.0±2.2 years, n = 2 576), and leatherbacks nested again after 1 065±682 days (or 2.9±1.8 years n = 535). Likewise, the proportion of turtles returning after one, two and three years was similar between the two species (Figure 7).

## Discussion

The prediction for this study was that both sea turtle species would benefit from protection in the iSimangaliso Wetland Park, because both species were under growing pressure from subsistence harvesting [39]. Leatherback eggs were taken on a large scale [39], while nesting loggerhead females were targeted with “carcasses strewn along the beach” [42]. Harvesting of both species ceased with the onset of active conservation and the proclamation of the coastal MPAs, which have now been in place for decades. The expectation was therefore that the two species should increase in abundance at a predictable continuous rate relative to reproductive output and (initial) population size.

Our data, however, suggested very different abundance trends in the two species. Both the index and monitoring areas showed a significant increase in the number of loggerhead nests over the 45 seasons (Figure 2). In contrast, the number of leatherback nests (in the index area) increased from 10 to 70 nests over the first decade, but since then has oscillated, and declined recently. Active conservation therefore seems to have facilitated a loggerhead population expansion but not an increase in the leatherback population.

The conservation success of the Maputaland leatherback population may, however, be relative. The maintenance of such a small population (of<100 females nesting.y^−1^) for three decades may be deemed a conservation success because many larger leatherback populations have collapsed recently despite protection [44]. Only populations from the North Atlantic (e.g. St Croix and Florida) have increased consistently over time [36], [37]. It appears therefore that iSimangaliso has been at least effective in maintaining a small population of leatherbacks nesting in the Western Indian Ocean, though it has not been able to facilitate a population increase in the long term.

The reason for these disparate responses between the species to conservation is not obvious, but four possible explanations are considered: (1) aspects of reproductive biology cause differences in reproductive output between the two species; (2) the leatherback population is increasing but the monitoring program is not capturing this trend (e.g. diffused nesting manifested as a range expansion or sex biased incubation impacting on the population); (3) the leatherback population has reached carrying capacity; or (4) there is differential offshore mortality countering localized conservation efforts [52].

The growth potential of a sea turtle population depends *inter alia* on the reproductive output per individual female [52], the incubation environment that determines both hatching/emergence success [52]–[54], population size [55], and sex ratios [56]. Reproductive output and emergence success were investigated for both species nesting in Maputaland [50]. Reproductive output was calculated as the number of nests per individual per season multiplied by the average number of eggs per clutch. The individual reproductive output per loggerhead female is lower than per leatherback female (ca. 389 eggs vs 699 eggs), but emergence success marginally favors loggerheads (at 78% vs 69% for leatherbacks). Nevertheless, hatchling production per female per season is considerably higher in leatherbacks (∼480 hatchlings) than in loggerheads (∼302 hatchlings). Given similar remigration periods in the two species, and lower age at maturity in leatherbacks (∼16 years vs. 36 years in loggerheads), population growth potential is higher in leatherbacks.

As the hatchling production *per se* does not explain the lack of leatherback population growth, the gender of the hatchlings produced may be a contributing factor to the differential population growth. There is currently some, but little information available on the loggerhead sex ratios [57], [58] and none for leatherbacks of Maputaland. The nesting distributions across the monitoring area are however well described (Figure 4), with very different habitat preferences for the two species [50]. These different habitat preferences result in different environmental incubation conditions, which may affect sex ratios [59]. The concentrated distribution of loggerhead nests means that such nests are more likely to develop under a limited range of environmental conditions than the broad range (and concomitant variation in temperature, dune morphology and vegetation) selected by leatherbacks (pers. obs). The higher spatial concentration of loggerhead nests resulted in maximum densities that were an order of magnitude higher (at 120±62 SD nests per km between 10–12 km south of the Kosi estuary) than in leatherbacks (at 11±8 SD nests per km at 48–50 km south of the Kosi estuary). Furthermore, within-season repeat nesting events (i.e. nest site fidelity) for leatherbacks are on average ca. 10 km apart, whereas loggerhead nests per individual are spread over less than 4.5 km [60]. Loggerheads become even more accurate with repeat nesting seasons, and nest sites of individual turtles are less than 1.5 km apart by the 5th nesting season [60]. Loggerhead recovery may thus have benefited from the higher abundance and spatial specificity (Figure 4), because the nest site fidelity of loggerheads might have resulted in the production of primarily females (during peak season) as suggested by [57], [58]. In contrast, leatherback nesting is widely distributed, with the higher nesting densities towards the southern, cooler end of the MPA. Cooler temperatures produce more males, so sex ratio may potentially be male-biased, or may have been male biased in the past. This notion is supported by the sex ratios of mature turtles caught in shark nets, which indicate a clear male bias for leatherbacks (M:F = 2∶1) and a more balanced sex ratio for loggerheads (at M:F = 1.4∶1; [61]). We therefore suspect that the production of female leatherbacks in South Africa may be limited by the dispersed, low-density nesting towards the southern extreme of the distribution, resulting possibly in a male bias. However, in South Africa, further research is needed to confirm sex bias as a reason for slow recovery in leatherback as was suggested for leatherbacks of the Huon Coast, Papua New Guinea [62].

The dispersed nesting distribution of leatherbacks may also compromise monitoring efforts if turtles do not return to the monitored area. Thorson et al. [63] evaluated the recapture potential of SA loggerheads and leatherbacks, and concluded (as did this study) that the recapture probability of loggerheads is higher (at 67%) than of leatherbacks (at 46%), possibly due to the restricted spatial distribution and early-evening emergence of loggerheads. They also investigated the recapture probability over time, and found that the loggerhead recapture probability is more consistent over time than that of leatherbacks. The majority (75–80%; [64]) of the nesting of both species takes place in SA, but the large distribution (∼900 km) and low density of leatherback nests, extending well into Mozambique [39], [49], complicates the accurate observation of leatherback turtles or their nesting events across the entire the population. Furthermore, increasing nesting numbers (or probability) may manifest as a “range extension” with increased distribution but constant density in the index area. If an area outside of the monitored area (which is∼330 km including monitoring programs in Mozambique) is preferred, only the “spillover” nesting may be captured by the SA monitoring program [63].

Low adult carrying capacity appears to be an unlikely reason for the lack of population growth in leatherbacks. Satellite tagging has indicated that this small population of leatherbacks uses both the Atlantic and Indian Ocean basin as foraging areas [65], and even though the carrying capacity in the Benguela Current has been altered for taxa such as sea birds (competing with seals and fisheries for food) [66], jellyfish have proliferated [67]. The body condition of females coming ashore to nest is excellent with an average size of∼1.6 m CCL, and above average clutch sizes and hatching success. Perault et al. [68] suggested this to be an indicator of good health status of adult females. Nesting space does not appear to be at a premium either; nesting and hatching success is generally good and exceed>70%. Nesting densities are currently also two to three orders of magnitude lower than, for example, in the world's largest leatherback rookery on the west African coast off Gabon [69], which shares a part of its foraging area with this population [69], [70]. The total number of clutches per annum produced in the Gabon rookery range from∼36 000–126 000 nests per annum. This equates to 83–292 nests.km^−1^ as opposed to only 3–19 nests.km^−1^ in iSimangaliso Wetland Park. There is also no evidence of leatherback nests being dug up by other turtles or significant beach predation (De Wet, NMMU, Unpublished data).

Another possible explanation for the apparent differential recovery between the two species is a disparate offshore mortality countering beach conservation actions [71], [72]. Little information is available for any of the post-hatchling or juvenile phases of either species and we therefore concede that some environmental factor or environmental carrying capacity at any of the immature size classes may limit the number of hatchlings returning as adults. It is possible that loggerhead and leatherback hatchlings do not end up in the same environment (i.e. Atlantic vs Indian Ocean basin), which may result in differential recruitment (into the adult population) [73]. Indeed, variability in oceanographic pattern causes high and variable mortality of leatherback hatchlings from New Guinea, which affects population growth rate in the area [74]. Similar processes may limit hatchling survival in the South African populations. However, as no information is available on the post-hatchling and juvenile distribution or survival of either species, there is no evidence of differential effects of currents and offshore conditions on the productivity of the two species.

In terms of human-induced threats, the largest estimated offshore threat to South African sea turtles is pelagic longlining [75]. This fishery started in the 1960s and operated at a relatively low level, but gained momentum in 1995 due to a joint venture fishing program between South Africa, Japan and Taiwan [76]. Bycatch monitoring between 2000 and 2005 suggested disproportionately high leatherback bycatches in this fishery [76]; loggerhead catches constituted 60.0% of all turtles, at a rate of 0.02 turtles per 1000 hooks, whereas leatherbacks were the second most frequently caught sea turtle species at 33.8% and a rate of 0.01 turtles per 1000 hooks. No information is available on the individual sizes of either turtle species nor on their origin, but it is likely that the majority originate from the South African rookeries as they were caught in the South African Exclusive Economic Zone (EEZ), with few other rookeries of either species in the vicinity. Although most turtles are reported to be released alive, no information is available on post-release survival of either species so some impacts have to be assumed. Given much smaller leatherback population size (1/5th to 1/10th), the longlining capture rates may constitute a much higher fishing mortality rate for leatherbacks than for loggerheads. Given the lack of data, improved data collection, both on catches (including morphometric information and genetic samples) and on post-release survival should be a high research priority.

Turtles are also caught in prawn trawl fisheries, bather protection nets off the east coast of KZN, as well extensive artisanal fisheries using a variety of gears, including gill nets, spears, or beach seines operated in the Mozambique Channel [75], [77]. The abundance of turtles caught in artisanal fisheries along the Mozambique Channel could overshadow commercial fishery catches, but catches have not been quantified [78], [79]. There are some data indicating that artisanal fishing pressure (including bather protection nets) appears to be a function of relative abundance without a significant bias per species [80]. This cannot be said for the other destructive commercial fishery; shallow-water trawling effort declined dramatically off KZN over the last decades due to estuarine degradation. More than 7 000 trawls were recorded from 1989 to 1992, dropping to less than 1 300 during 2003–2006 and to an average of 25 trawls per year during 2007–2011 (Fenessy, ORI, Unpublished data). Extrapolating from an observed catch rate of 0.13 loggerheads per trawl, catches might have been as high as 230 ind.y^−1^ in the early 1990s, but only 40 ind.y^−1^ in 2003–2006 (De Wet, NMMU, Unpublished data). The observed catch rate for leatherbacks was 0.0008 individuals per trawl, so approximately 1.4 ind.y^−1^ caught in the 1990s and 0.2 ind.y^−1^ caught post 2003 (De Wet, NMMU, unpublished data). This indicates a species bias but also a marked reduction in trawl impacts on loggerheads off KZN, and presumably a large reduction in mortality rates, even though turtle (or general bycatch) reduction devices (TEDs/BRDs) were not routinely employed in this fishery [81]. The decline in the prawn trawl fishery may have contributed to the recovery of loggerheads while longlining might have contributed to the demise of leatherbacks.

The temporal trend in the leatherback to loggerhead ratio in the South African rookery (Figure 3) provides some evidence to support the effects of fisheries on these populations. Leatherbacks started to decline relative to loggerheads at the expansion of the longline fishery (1990–1995), with loggerhead abundance peaking only after prawn trawling effort declined. The nesting habitat and females are well protected, but beach protection contributed to the expansion of the population only at inception by removing the local harvesting pressures. In reality, the spillover usage envisaged continued over time but not only in subsistence fisheries. It appears that the commercial fishery pressure was sufficient to suppress the population expansion of both species at various times.

The issues raised in this paper (i.e., coastal conservation, habitat preferences and sex ratios, and offshore pressures) highlight the difficulties of predicting the long-term success of one particular conservation approach to migratory species, especially if only limited population components (nesting females/nests) are adequately protected and monitored. At the very least, adult males should be monitored, possibly via paternal identification from hatchling DNA. Additional research priorities could include the post-hatchling distribution, particularly to identify developmental habitats for these two species. This will facilitate both the understanding of population ecology as well as the relative threats per size class.

In reviewing the responses of the two sea turtle species to long-term conservation, we concluded the following: first, highly migratory species such as loggerhead can benefit from conservation in coastal MPAs. Leatherbacks did not respond as predicted, but managed to maintain a stable population over time despite the small size. Second, reproductive output alone is no guarantee for population growth especially in species with temperature-dependent sex determination. The initial population size and site specificity resulting in higher density nesting seems to have favored loggerhead population growth (even if it was just to facilitate monitoring). The comparatively high reproductive output per individual leatherback female did not seem to facilitate population expansion. Alternative strategies to foot patrols and coastal MPAs should be considered for this rookery to better survey leatherback abundances. It appears to be sub-optimal to protect and monitor species in areas selected by default (e.g., overlap in distribution with another species, in this case with loggerhead turtles) as the area may include only marginal nesting areas. Third, (despite these monitoring short-comings) in the case of oceanic migrants, offshore conservation should supplement coastal conservation efforts. Both loggerhead and leatherback turtles are vulnerable to fisheries; loggerheads particularly to trawling and artisanal (gillnet) fisheries and leatherbacks to industrial longline fisheries. Population growth rate could increase for both species if offshore threats (especially in the larger size classes) are reduced. In conclusion, this research suggests that MPAs can be effective in local protection of high density or sensitive life history stages, but they are inadequate as a sole conservation measure in oceanic migrants, especially in the long term with changing pressures. Further, differences in recovery between species have to be interpreted with caution, as the cause for disparate population trends even in migratory species may lay within the MPA. Careful quantitative research is necessary to identify the causes of recovery (or the lack thereof) to ensure appropriate conservation strategies.

## Supporting Information

Figure S1 Time-trajectories of estimated counts for loggerheads (upper panels) and leatherbacks (lower panels) for the Monitoring Area (left panels) and Index Area (right panels). Results are shown for three alternative models.
(TIF)
